# Histologic and Histomorphometric Analysis of Bone Regeneration with Bovine Grafting Material after 24 Months of Healing. A Case Report

**DOI:** 10.3390/jfb9030048

**Published:** 2018-08-08

**Authors:** Renzo Guarnieri, Fabrizio Belleggia, Patricia DeVillier, Luca Testarelli

**Affiliations:** 1 Department of Dental and Maxillofacial Sciences, School of Dentistry, University La Sapienza, 00100 Rome, Italy; lucatestarelli@gmail.com; 2 Private Periodontal Implant Practice, 00100 Rome, Italy; fabriziobelleggia@gmail.com; 3 Department of Oral and Maxillofacial Pathology, University of Alabama at Birmingham, Birmingham, AL 35294, USA; Patriciadevillier@gmail.com

**Keywords:** histology, histomorphometry, anorganic bovine bone mineral matrix, sinus augmentation

## Abstract

Anorganic bovine bone mineral matrix (ABBMM) has been reported to have osteoconductive properties and no inflammatory or adverse responses when used as grafting material in sinus augmentation procedures. However, controversy remains in regard to degradation rate of ABBMM. The aim of this study was to histologically and histomorphometrically evaluate the degradation of ABBMM in human bone samples obtained in one patient 24 months after sinus augmentation. Materials and Methods: The histologic and histomorphometric analysis was performed by means of light microscopy in three specimens harvested from the same patient, Results: After 24 months the tissue pattern appeared to be composed of residual particles, some in close contact with the newly formed bone, others separated by translucent areas and osteoid tissues. Newly-formed bone presented different levels of maturation and numerous osteocytes, with greater numbers in bone closer to the grafted particles (27.3% vs. 11.2%, *p* < 0.05). The histomorphometric analysis showed mean values of 40.84% newly-formed bone, 33.58% residual graft material, 23.84% marrow spaces, and 1.69% osteoid tissue, Conclusions: Even though ABBMM underwent considerable resorption, a great amount of residual grafting material was still present after two years of healing following sinus augmentation. This study confirms that the bovine grafts can be classified as long-term degradation materials.

## 1. Introduction

The final goal of any bone grafting procedure should be the achievement of 100% living and reactive tissue able to undergo a sustained state of remodeling. Autogenous bone has always been considered the gold standard for grafting procedures because of the reproducible healing mechanism of osteogenesis, osteoinduction, and osteoconduction [1]. However, autogenous bone also has disadvantages, including limited amount of available graft material, an additional surgical site, donor site morbidity, and the requirement of general anesthesia for extraoral bone harvesting [2,3]. Moreover, when used in sinus augmentation, autogenous bone has been reported to be rapidly resorbed and this might compromise implant placement [4].

In order to overcome these disadvantages, xenogenic bone has been proven as alternative graft material in bone regeneration [5,6].

Anorganic bovine bone mineral matrix (ABBMM) is by far the most commonly used and researched xenograft. Most of the available ABBMM consist of deproteinized and sterilized bovine bone. Such matrices provide a scaffold for cells migration and are involved in the osseointegration and remodeling processes [7].

Contrasting data are present in the literature regarding whether ABBMM is completely degradable and whether the presence of residual graft particles could interfere with the healing process of regenerated sites [8]. This discrepancy may be attributed to differences in the type of study (animal vs. human studies), surgical approach, biopsy technique, or histological evaluation method [9]. Non-resorption might result in shielding of the newly-formed bone from physiological stresses necessary for further remodeling and maturation. Moreover, it could also influence or interfere with the osseointegration process of dental implants and bone-to-implant contact [1,10]. Residual ABBMM granules around the dental implant body could represent a locus minoris resistentiae in case of peri-implant infection [11]. Few human histologic reports on ABBMM resorption rate over time are available in the literature [12,13,14,15,16]. Histological studies have shown that ABBMM presents a markedly faster resorption in the initial period (3/6 months) after graft insertion [11,17,18,19], but it is slowed down in subsequent time periods [16,20]. Contrasting results are reported in literature also on the biological interactions occurring at the bone–ABBMM interface [21]. Once the graft particles incorporation in the bone create a dense and hard tissue network they act similar to the host bone and provide appropriate biologic support [22,23,24,25]. On contrary, the presence of multinucleated cells and osteoclastic activity surrounding the residual particles suggests that the bone remodeling process may be negatively influenced [26,27,28,29].

It is quite important to understand the effect of residual graft particles at different time periods and if there is any interference with the natural bone processes that might possibly affect the prognosis in human.

For that reason, we investigated the biopsies taken from one patient after 24 months of maxillary sinus augmentation by using ABBMM. The aim of this study was to obtain histomorphometric measurements of newly-formed bone, marrow spaces, residual biomaterial particles and number of osteocytes being present.

## 2. Materials and Methods

A 50-year-old female with a monolateral maxillary edentulism involving the premolar/molar regions underwent a sinus augmentation procedure in January 2016. The preoperative mean height of the subantral bone was <4 mm. The patient was a non-smoker and had a non-contributory medical history. One-hundred percent of ABBMM was used in the surgical procedure. The patient, who was previously accepted in a study approved by the Ethical Committee of La Sapienza University, Roma; (reference no. 4597), provided written informed consent for all procedures. The study was conducted according to the principles embodied in the Helsinki Declaration for biomedical research involving human.

A thorough preoperative evaluation was performed, including the study of mounted diagnostic cast and diagnostic wax-up. Radiographic examination included both intraoral and computerized tomography. Preoperative medications included amoxicillin, and 1 g twice a day of clavulanic acid (NeoDuplamox, Procter and Gamble), starting one day prior to surgery and continuing until eight days post-surgery. Patient was asked to rinse with 0.2% chlorhexidine gluconate the day of surgery and twice a day for 14 days after the procedure.

Under local anesthesia, a crestal incision was made slightly toward the palatal aspect and throughout the entire length of the edentulous segment, supplemented by buccal releasing incisions mesially and distally. Full thickness flaps were elevated to expose the alveolar crest and the lateral wall of the maxillary sinus. A trap door was made in the lateral sinus wall using a round bur under sterile saline solution irrigation. The door was rotated inward and upward. The sinus membrane was elevated with curettes of different shapes until it became completely detached from the lateral and inferior wall of the sinus. The ABBMM (MinerOssX, BioHorizons, Birmingham, AL, USA) was mixed with venous blood and packed carefully in the sinus cavity. MinerOssX is a natural cancellous (spongiosa) and cortical bovine bone matrix with 250–1000 μm particles distribution size. It is produced by a chemical removal of organic components, has 75–80% porosity and a crystal size of approximately 10 μm [30].

The mucoperiosteal flap was then re-positioned and sutured with multiple horizontal mattress sutures. Sutures were removed two weeks after surgery. Postsurgical visits were scheduled at monthly intervals to check the course of healing. The sinus was allowed to heal for 24 months, and then three implants were placed into the grafted area.

### Histologic Analysis

Twenty-four months after maxillary sinus augmentation, three bone cores were harvested, before implants placement, using a 3.5 mm diameter trephine under cold (4–5 °C) sterile saline solution irrigation. The bone specimens were immediately fixed in 10% buffered formalin and embedded in a glycolmethacrylate resin. After polymerization, specimens were sectioned along their longitudinal axis to a thickness of 70 microns (plastic microtome, RM 2265, Leica, Buffalo Grove, IL, USA). Slides were stained with Trichrome and examined using an Olympus B51 microscope (Olympus America, Lake Success, NY, USA). The core area of every specimen was chosen for histomorphometric analysis. Images were captured with a Q-Imaging camera, (Retiga R1™ CCD camera, 32-0013B-157, 12-bit color, Surrey, BC, Canada) and area fraction percentages of every component in was measured automatically using Bioquant^®^ image analysis software (R&M Biometrics, Nashville, TN, USA). To evaluate bone quality, histomorphometric measurements were recorded according to the nomenclature approved by the American Society of Bone and Mineral Research, and analyzed by a blinded researcher using Ky Plot 2.0 software (Informer Technologies, Inc., New York, NY, USA).

## 3. Results

**Figure 1:**
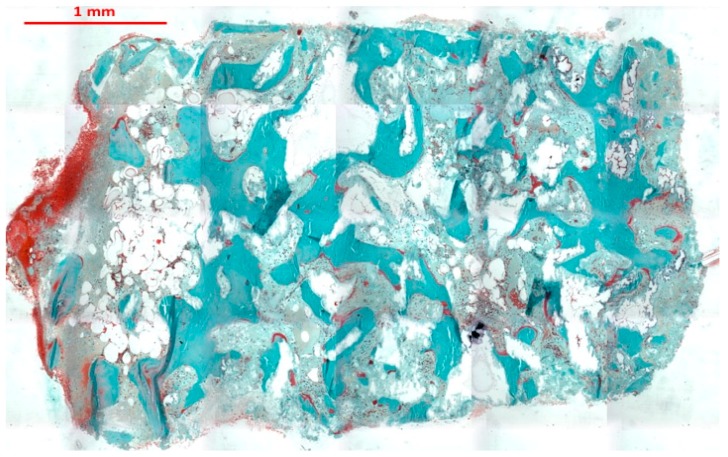
Panoramic histological image of bone core biopsy taken after 24 months from maxillary sinus augmentation (trichrome stain × 5). Residual ABBMM particles surrounded by vital bone which presents different levels of maturation.

Microscopic examination of processed bone core specimens showed newly-formed bone in close contact with ABBMM particles (Figure 1).

**Figure 2:**
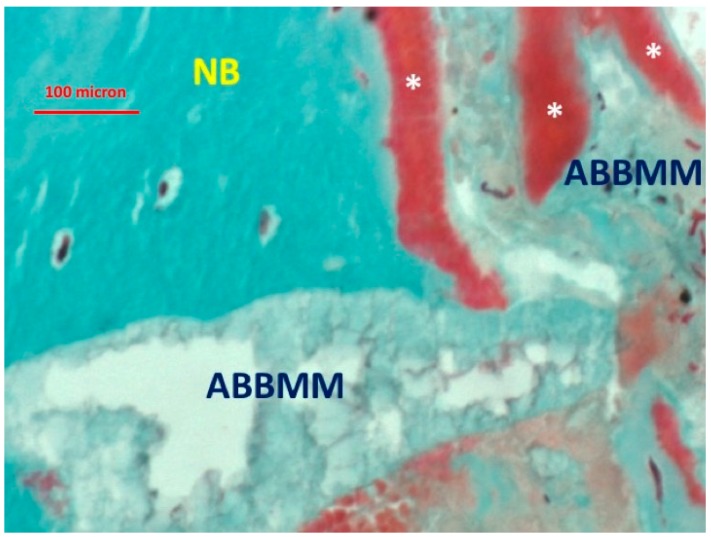
Presence of non-mineralized osteoid matrix (*) at the interface with the ABBMM (trichrome stain ×20).

**Figure 3:**
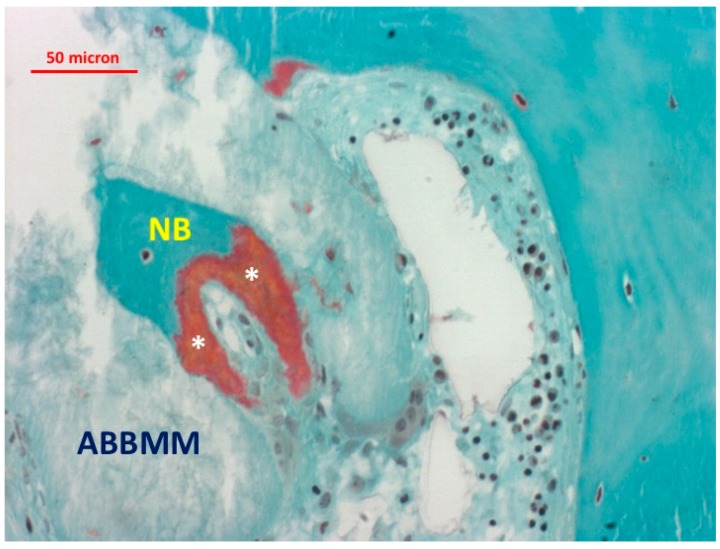
Presence of non-mineralized osteoid matrix (*) at the interface with the ABBMM (trichrome stain ×20).

Most of the graft particles were surrounded by newly-formed bone. In some areas, the graft particles were in contact with marrow spaces. The presence of non-mineralized matrix (osteoid seam) was also observed at the interface with the ABBMM (Figure 2 and Figure 3).

**Figure 4:**
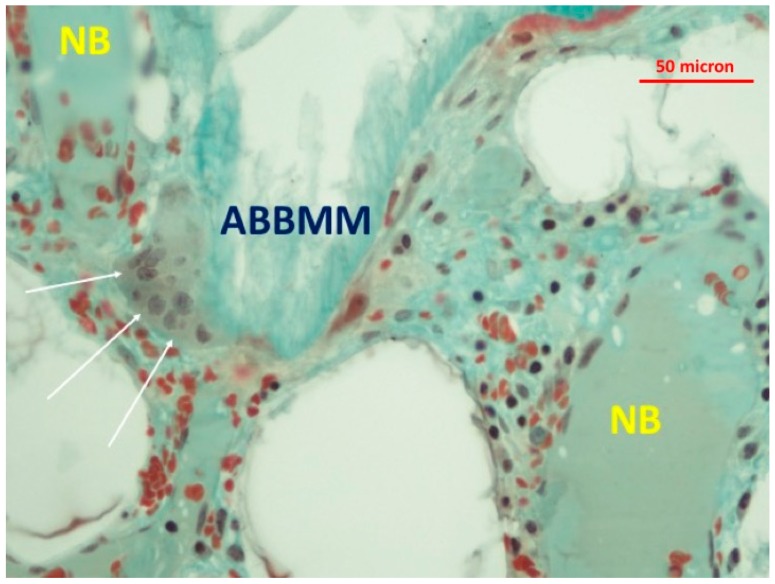
Presence of multi-nucleated cells (white arrows) at the interface between the biomaterial particles (ABBMM) and newly-formed bone (NB) (trichrome stain ×20).

**Figure 5:**
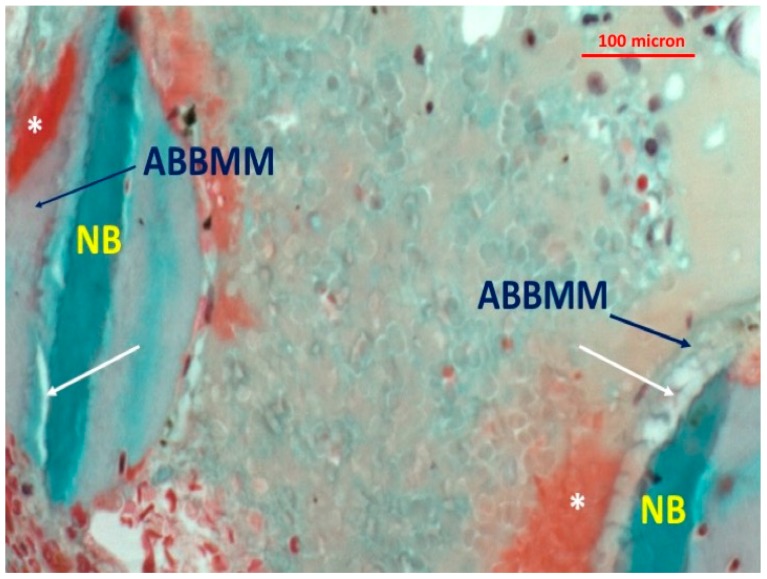
Presence of translucent areas (white arrows) at the interface between the biomaterial particles (ABBMM) and newly-formed bone. Non-mineralized osteoid matrix (*) (trichrome stain ×40).

**Figure 6:**
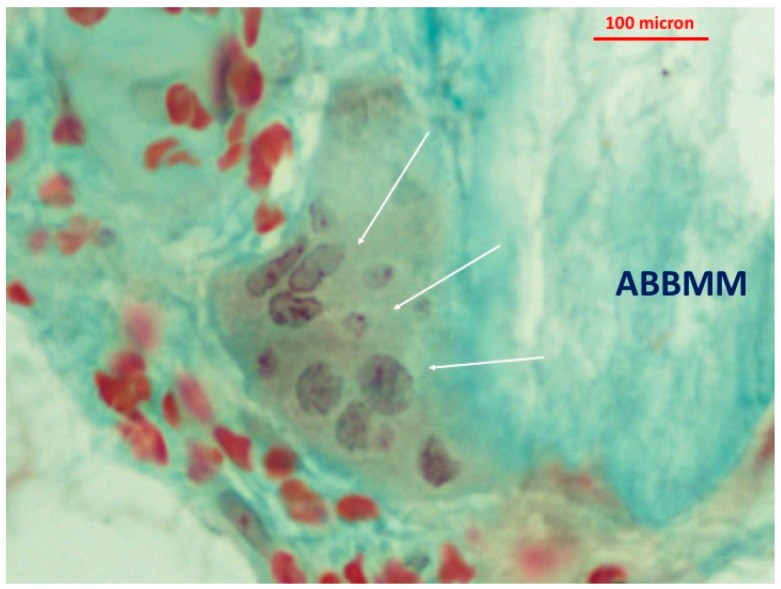
Higher magnification of Figure 4 showing multi-nucleated cells in close approximation and in contact with ABBMM surface.

The newly-formed bone abutting the graft particles showed viable bone and lacunae with osteocytes. Little granulocytic infiltrate was present in the bone marrow spaces. At higher magnification, a few multi-nucleated cells at the interface between the biomaterial particles and new formed bone were detected (Figure 4, Figure 5 and Figure 6). In addition, many translucent areas at the interface between the biomaterial particles and newly-formed bone and osteoid tissue were found (Figure 5). Histomorphometric data are reported in Table 1.

## 4. Discussion

It is important to understand the process of new bone formation and remodeling during early and late healing phases at sites grafted with ABBMM. The current study presents histomorphometric measurements of newly-formed bone, marrow spaces, residual biomaterial particles, and osteocytes in biopsies taken from one patient after two years of maxillary sinus augmentation by using ABBMM. It was observed that the amount of residual graft material was 33.58%. The results also showed a mean value of 40.84% newly-formed bone which is accordance to the previously reported sinuses grafted with ABBMM [21].

On the other hand, bone sample harvested from one extraction socket regenerated with the same ABBMM showed a mean value of 26.85% newly-formed bone after six months [27]. It is difficult to make a direct comparison between these two analyzes due to different defect architecture as wells as new bone formation, vascularization and graft particles degradation patterns. In addition, other study suggests that new bone formation increases over time in sites grafted with ABBMM [29]. More specifically, the new bone formation after sinus floor augmentation using ABBMM was about 36% at 6 months [30], and about 42% at 14 months [31]. Moreover, compared to 12 and 48 months of healing histomorphometric data at nine years showed a newly-formed bone increase of 18.45%, and of 8%, respectively [16].

Also the degradation time and ultimate fate of many commercially available bovine grafting materials at various grafted sites is also not fully understood. There are many variations among the tested models [13,32,33,34], cell types [11,34] and the histological preparation methods [9]. While it is generally accepted that graft particles undergo resorption by osteoclasts [15,34,35,36], it is documented that multinucleated cells are also present on the surface of the material [7,8,9,10,11,12,13,14,15,16,17,18,19,20,21,22,23,24,25,26,27,28,29,30,31,32,33,34,35,36,37,38,39]. Its hard to distinguishing between osteoclasts or macrophage polykaryons and if these cells are active osteoclasts, nonactive/impaired osteoclasts, giant cells or macrophages/monocytes undergoing fusion [40].

In order to better understand the degradation process, it is important to identify the type of cells surrounding the ABBMM particles. The histologic sections from the present study demonstrated multi-nucleated cells in close approximation and in contact with new bone while the osteoblastic lineage formed new bone onto the ABBMM surface (Figure 6). Such a histologic pattern has been previously defined as “functional coupling in the bone metabolic unit” [41]. It is suggested that the multinucleated giant cells observed in the present study could have the function of macrophages polykaryons. They probably “clean” the graft particles surface from the degradation products and therefore prepare the conditions for deposition of newly-formed bone [41].

In sites grafted with ABBMM after the first initial healing period (3–6 months), the osteoclastic activity in the microenvironment around the biomaterial particles could be inhibited by progressive increase of Ca^2+^ ions concentration [16]. It has been documented that acid secretion by osteoclasts causes mineral release from the substratum surface, which leads to an increase in Ca^2+^ ions in this compartment [16]. This, in turn, slows down the osteoclastic activity [42]. Ultrastructural analysis by backscattered electron imaging analysis showed a higher Ca/P ratio in the residual the interface compared with new bone [43]. This suggests that there may be a gradual diffusion of Ca^2+^ ions from the biomaterial into the newly-forming bone at the interface as part of the biomaterial’s resorption process. The presence of many translucent area at the interface between the biomaterial particles and newly formed bone and osteoid tissue found in the present study may represent the lytic process of the graft (Figure 5).

The degradation process of bovine-derived bone depends on the production process that can cause variations in physicochemical properties, hydrophilicity, and viscoelasticity [44,45]. More specifically, the high temperature sintering method leads to increased mineral crystalline size, which imparts a lower degradation rate if compared to the low temperature and chemical treatment methods.

The degradation process is influenced also by pores morphology, degree of porosity, pores’ interconnections, and granule size distribution [46]. A decrease in pore connectivity could influence the possibility that a greater number of osteoblasts can penetrate the porous structure. In addition, also the degree of angiogenesis and the resulting flux of nutrient and of oxygen could be lower [47].

Another interesting data documented in the present study is related to the higher mean of osteocytes/area measured in the bone around the grafted particles compared to those found in the bone at a distance from the particles. The complex biologic function of osteocytes is still to be elucidated. It was suggested that osteocyte play a relevant role in the bone homeostasis and remodeling [48]. Osteocytes may produce signals to control osteoblast and bone lining cell functions, and thereby regulate bone modeling and help with new bone formation [49,50]. In vitro studies showed that osteocytes are negative regulator of osteoclast activity and may play a major role in triggering local bone remodeling [51]. In particular, it has been documented that osteocyte apoptosis triggers a bone remodeling response, while the neighboring non-apoptotic osteocytes are a major source of pro-osteoclastogenic signals. Moreover, both the apoptotic and osteoclast signaling osteocyte populations are localized in a spatially and temporally restricted pattern consistent with the targeted nature of remodeling response [52]. Lacking sufficient live osteocytes possibly leads to inefficiency in the remodeling activity. The higher number of osteocytes/area found in the present study in bone around the grafted particles, compared to bone at a distance from the graft, could be considered a “bone strategy” to overcome the absence of functional syncytium inside the biomaterial particles [52,53].

Most of the histological analysis after sinus augmentation report results at implant placement time during 6 to 9 months post grafting. Even though the present study is limited by the small sample size of three biopsies taken from one and the same patient, it still gives a general idea regarding the degradation process of ABBMM following sinus lift procedure after 24 months of healing. The histologic and histomorphometric analysis showed that a great amount of the residual grafting material was still present but it was still less than the newly-formed bone. More studies are required to understand the new bone formation patterns at different time points and in various grafted defects.

## 5. Conclusions

The present histologic and histomorphometric analysis was aimed to compare histomorphometric measures for newly-formed bone, marrow spaces, biomaterial particles remnants, and number of osteocytes embedded in both trabecular bone and bone tissue near the ABBMM. At two years healing time following sinus augmentation ABBMM underwent significant degradation. The tissue pattern appeared composed by residual ABBMM particles in close contact to the newly-formed bone and to osteoid tissue.

## Figures and Tables

**Table 1:** Histomorphometric data (mean ± SD).

Column	Mean	SD
Tt. Tissue Area.	7.205	(1.8)
Tt. Area of Bone	1.952	(0.5)
Tt. Area of Bone Graft	3.103	(0.9)
Tt. Osteoid Area	0.095	(0.03)
Tt. Connective Tissue Area	2.054	(0.8)
%. Marrow spaces/Tt. Area	23.84	(4.6)
%. Bone/Tt. Tissue Area	40.84	(3.3)
%. Graft/Tt. Tissue Area	33.59	(2.8)
%. Osteoid/Tt. Tissue Area	1.697	(0.4)
